# Supplementary material for: Multiple ageing effects on testicular/epididymal germ cells lead to decreased male fertility in mice
Source: Commun Biol. 2024 Jan 4;7:16. doi: 10.1038/s42003-023-05685-2 (PMC10766604; doi:10.1038/s42003-023-05685-2)
Supplement: Supplementary file 2 — Supplementary Information [file 42003_2023_5685_MOESM2_ESM.pdf]

Supplementary Figures

Multiple ageing effects on testicular/epididymal germ cells lead to decreased male fertility in mice.

Tsutomu Endo[1,2,3,4,*], Kiyonori Kobayashi[2,5], Takafumi Matsumura[2,6], Chihiro Emori[2], Manabu Ozawa[7], Shimpei Kawamoto[2], Daisuke Okuzaki[2], Keisuke Shimada[2], Haruhiko Miyata[2], Kentaro Shimada[2,6], Mayo Kodani[2,6], Yu Ishikawa-Yamauchi[7,8], Daisuke Motooka[2], Eiji Hara[1,2,5,9], and Masahito Ikawa[1,2,6,7,9,*]

Supplementary Fig. 1

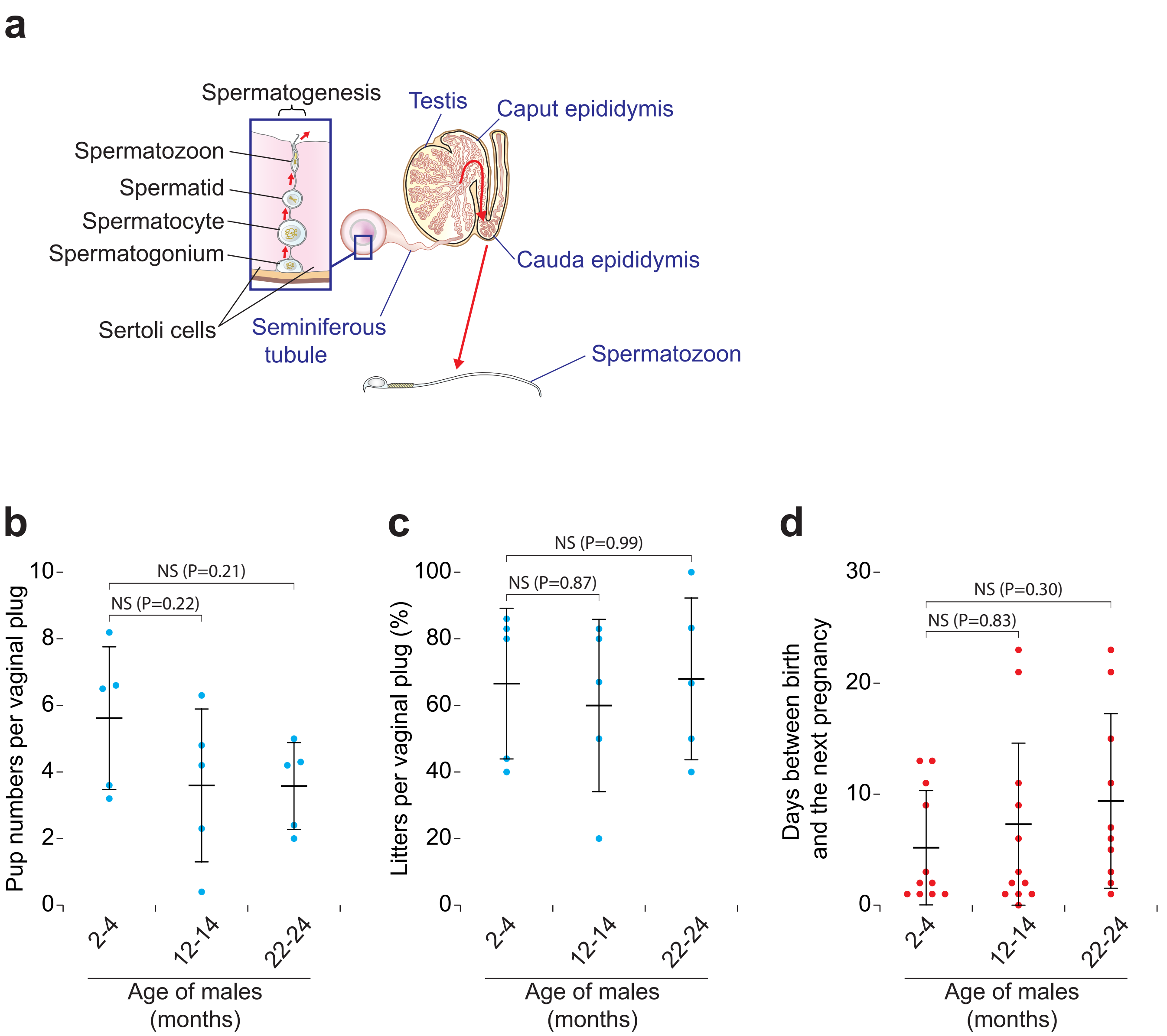

Supplementary Fig. 1 Mating frequency is not significantly changed in males with age.

(**a**) Structure of the mouse testis comprising seminiferous tubules and epididymis. In any given seminiferous tubule cross-section, one observes germ cells at different steps in their development into spermatozoa. Spermatozoa are released into the lumen of the seminiferous tubules and acquire their motility and fertility as they migrate through the head (caput), body (corpus), and distal (cauda) epididymis.

(**b**) Pup numbers per vaginal plug. Females (n = 30) were mated with males (n = 5; blue dots) at 2-4, 12-14, or 22-24 months of age. NS, not significant ($P > 0.05$; Dunnett's test).

(**c**) Litters per vaginal plug (%). Females (n = 30) were mated with males (n = 5; blue dots) at 2-4, 12-14, and 22-24 months of age. NS, not significant ($P > 0.05$; Dunnett's test).

(**d**) Days between birth and the next pregnancy. Each red dot represents the days between birth and the next pregnancy when females (n = 30) were mated with males (n = 5) at 2-4, 12-14, and 22-24 months of age. NS, not significant ($P > 0.05$; Dunnett's test).

Supplementary Fig. 2

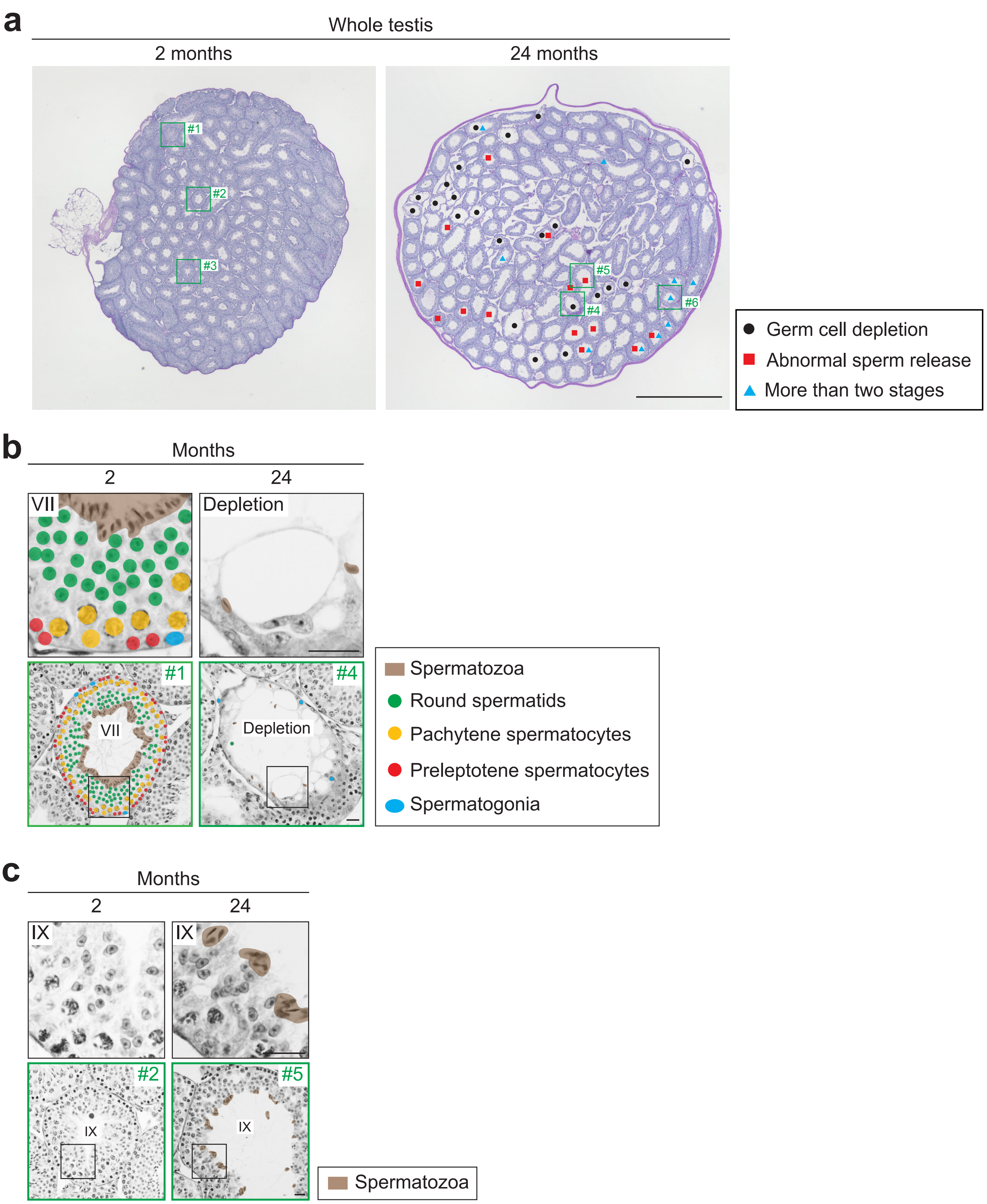

Supplementary Fig. 2 Representative whole testis cross-section and the tubule cross-section with abnormal sperm release in aged males.

(**a**) Whole testis cross-sections in males at 2 (*Left*) and 24 (*Right*) months of age, stained with hematoxylin and periodic acid-Schiff (He-PAS). Individual images are stitched together for an entire testis. Green boxed regions (#1-6) are enlarged in Fig. 2e, g, and j. Black dots, tubule cross-sections showing germ cell depletion. Red squares, tubule cross-sections showing abnormal sperm release. Blue triangles, tubule cross-sections showing more than two seminiferous stages. Scale bar, 1 mm.

(**b**) Grayscale versions of testis tubule cross-sections shown in Fig. 2e. Dots, type A spermatogonia (blue), preleptotene spermatocytes (red), pachytene spermatocytes (yellow), and round spermatids (green). Brown areas, spermatozoa. Scale bars, 20 μm.

(**c**) Grayscale versions of testis tubule cross-sections shown in Fig. 2g. Brown areas, spermatozoa. Scale bars, 20 μm.

Supplementary Fig. 3

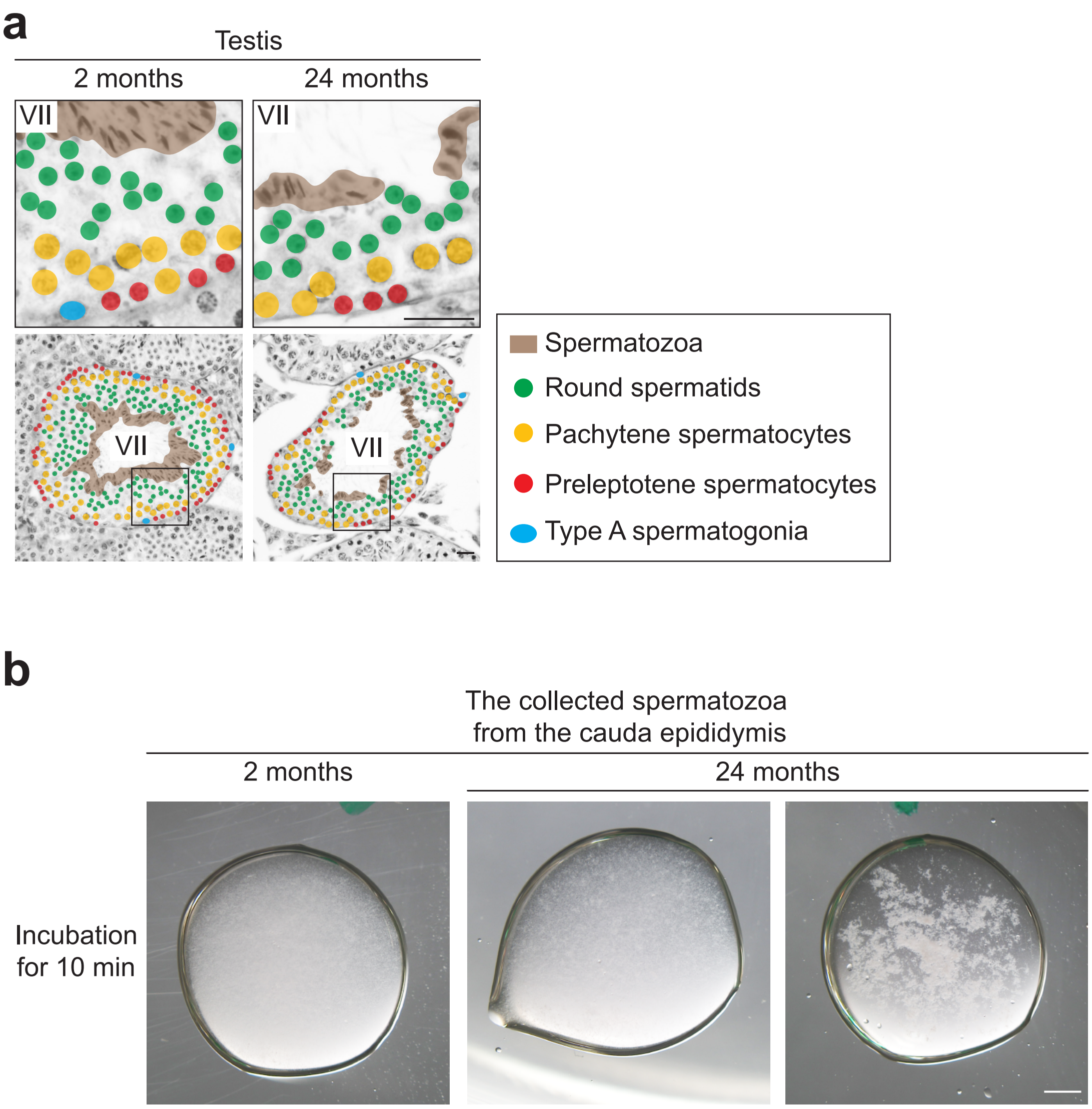

Supplementary Fig. 3 Numbers of testicular germ cells and epididymal spermatozoa are decreased with age.

(**a**) Grayscale versions of testis tubule cross-sections shown in Fig. 3a. Dots, type A spermatogonia (blue), preleptotene spermatocytes (red), pachytene spermatocytes (yellow), and round spermatids (green). Brown areas, spermatozoa. Scale bars, 20 μm.

(**b**) The collected spermatozoa from the cauda epididymis, in males at 2 (*Left*) and 24 (*Middle* and *Right*) months of age. The spermatozoa were incubated for 10 min in TYH medium. Scale bar, 1 mm.

Supplementary Fig. 4

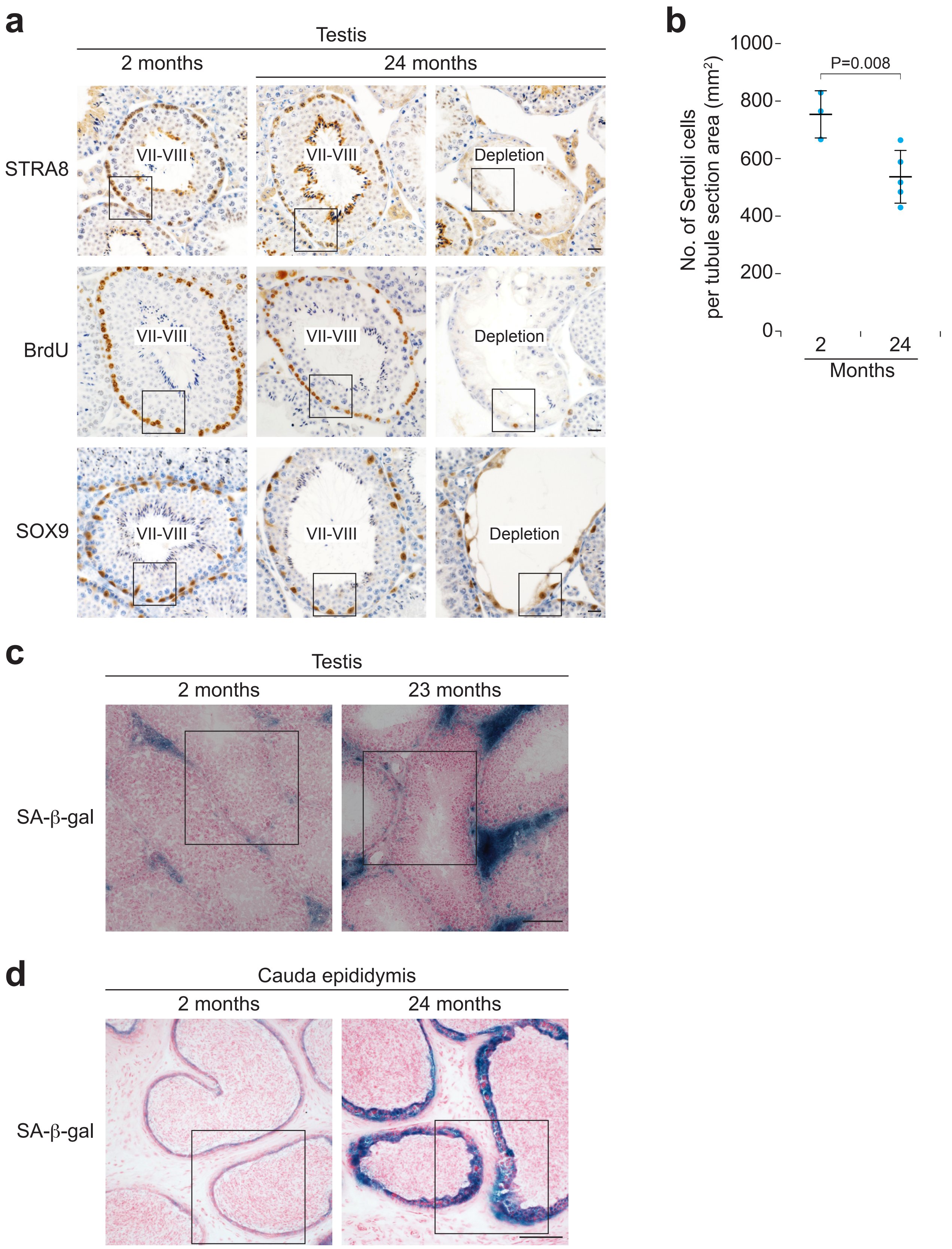

Supplementary Fig. 4 Key protein marker expressions related to spermatogonial differentiation, meiotic initiation, and Sertoli cells in aged testes.

(**a**) Immunostaining for STRA8, BrdU, and SOX9, with hematoxylin counterstain, on testis cross-sections of males at 2 (*Left*; a stage VII-VIII tubule) or 24 (*Middle*, a tubule in stages VII-VIII; *Right*, a tubule showing germ cell depletion) months of age. Arrows, Sertoli cells. Arrowheads, type A spermatogonia (white) and preleptotene spermatocytes (black). Black boxed regions are enlarged in Fig. 2a. Scale bars, 20 μm.

(**b**) Number of Sertoli cells in stage VII-VIII tubule cross-sections per tubule section area (mm^2). Blue dots, biological replicates of males at 2 (n = 3) and 24 (n = 5) months of age. P = 0.008 (one-tailed *t* test).

(**c**) Senescence-associated β-galactosidase (SA-β-gal) staining, with Nuclear Fast Red counterstain, on testis cross-sections of males at 2 (*Left*) or 23 (*Right*) months of age. Black boxed regions are enlarged in Fig. 4e. Scale bars, 100 μm.

(**d**) Senescence-associated β-galactosidase (SA-β-gal) staining, with Nuclear Fast Red counterstain, on cauda epididymis tubule longitudinal-sections of males at 2 (*Left*) or 24 (*Right*) months of age. Black boxed regions are enlarged in Fig. 4g. Scale bars, 100 μm.

Supplementary Fig. 5

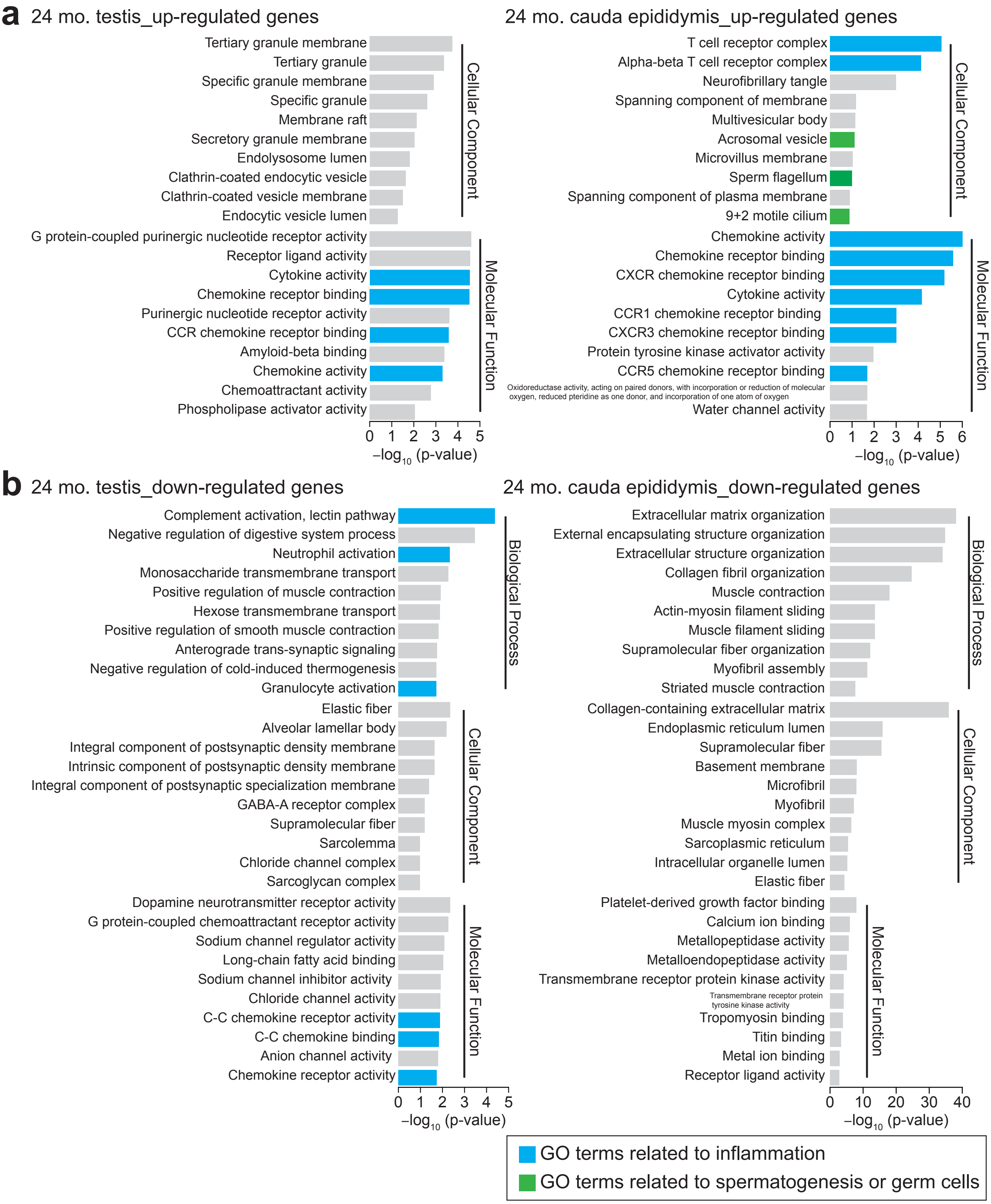

Supplementary Fig. 5 Gene Ontology (GO) terms on RNA-Seq analysis in aged testes and epididymides.

(**a**) Gene Ontology (GO) terms (cellular component and molecular function) of up-regulated genes (fold change ≥ 2) on RNA-Seq data in testes (*Left*) or cauda epididymides (*Right*) of males at 2 (n = 5) vs. 24 (n = 5) months of age. The top 10 enriched terms are displayed based on the -log10 (P-value). Y-axis, -log10 (P-value). Blue graphs, GO terms related to inflammation. Green graphs, GO terms related to spermatogenesis or germ cells.

(**b**) Gene Ontology (GO) terms (biological process, cellular component, and molecular function) of down-regulated genes (fold change ≤ -2) on RNA-Seq data in testes (*Left*) or cauda epididymides (*Right*) of males at 2 (n = 5) vs. 24 (n = 5) months of age. The top 10 enriched terms are displayed based on the -log10 (P-value). Y-axis, -log10 (P-value). Blue graphs, GO terms related to inflammation.

Supplementary Fig. 6

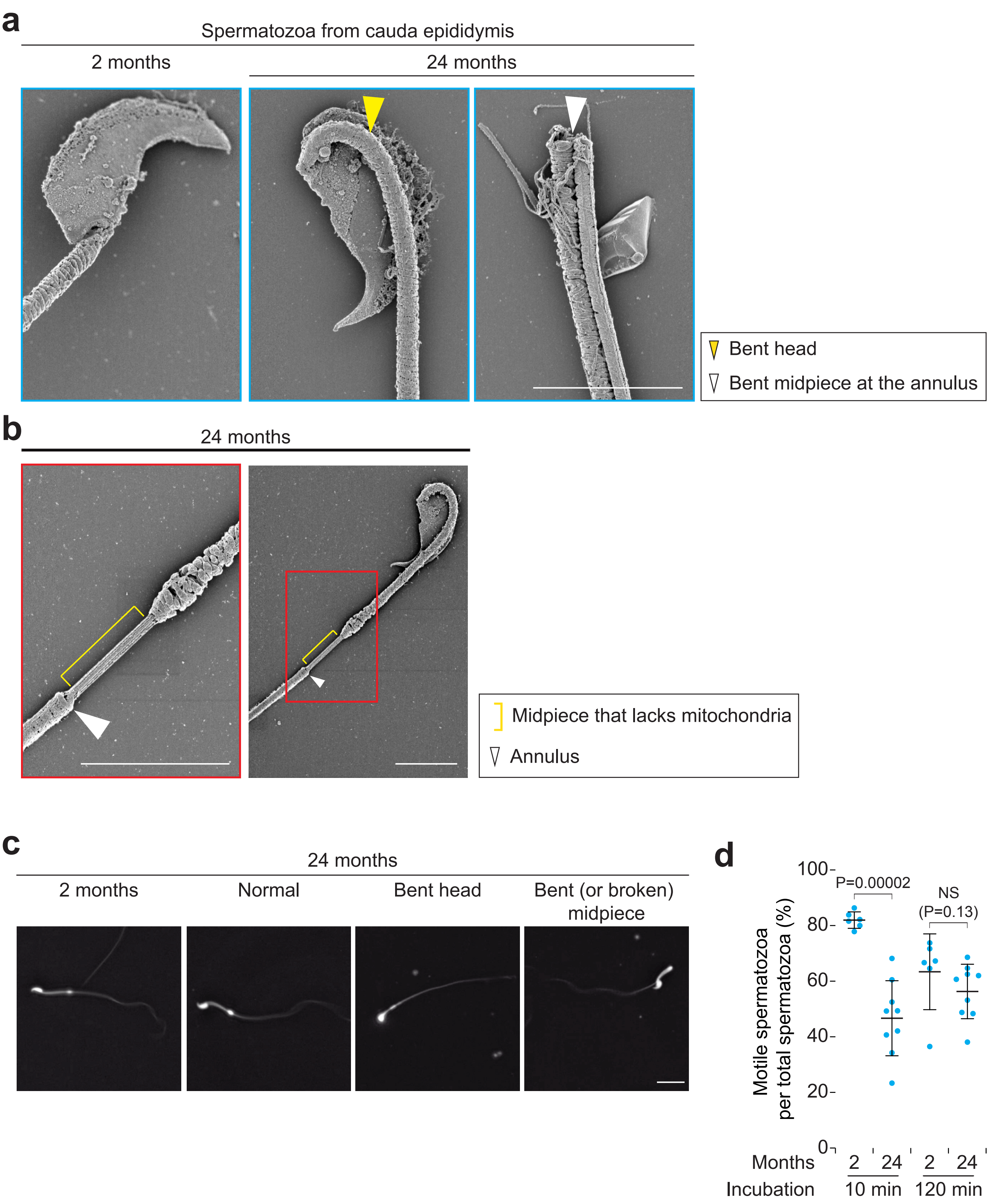

Supplementary Fig. 6 Spermatozoa in aged epididymides show morphological abnormalities and decreased motility.

(**a**) Ultrastructural analysis of cauda epididymal spermatozoa, in males at 2 (*Left*) and 24 (*Middle* and *Right*) months of age, by scanning electron microscopy (SEM). Blue panels in Fig. 6c are enlarged. Arrowheads, bent head (yellow) and bent midpiece at the annulus (white). Scale bars, 5 μm.

(**b**) Ultrastructural analysis of cauda epididymal spermatozoa, in males at 24 months of age, by scanning electron microscopy (SEM). Yellow bars, midpiece that lacks mitochondria. Arrowheads, annuli. *Left* panel enlarges the red boxed region in *Right* panel. Scale bars, 5 μm.

(**c**) Representative single frame images captured by a high-speed camera, for the analysis of flagellar bending patterns of spermatozoa, shown in Fig. 6d, in males at 2 (*Far left*) and 24 (*Center left*, normal; *Center right*, bent head; *Far right*, bent or broken midpiece) months of age. Scale bars, 20 μm.

(**d**) Motile spermatozoa per total spermatozoa (%), at 10 min and 120 min after incubation, in males at 2 and 24 months of age. Error bars, mean ± SD. Blue dots, biological replicates of males at 2 (n = 6) and 24 (n = 9) months of age. P = 0.00002 (one-tailed *t* test). NS, not significant (P > 0.05; one-tailed *t* test).

Supplementary Fig. 7

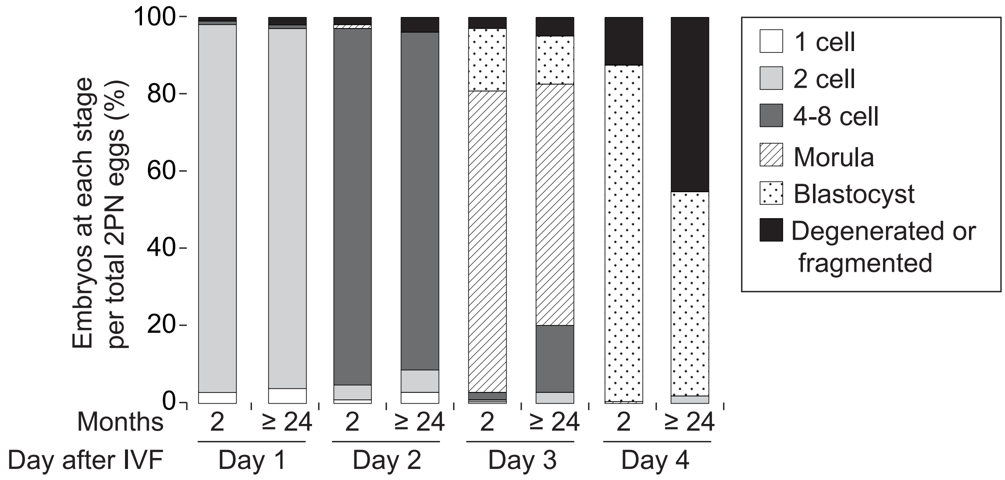

Supplementary Fig. 7 Spermatozoa in aged epididymides adversely affect early embryonic development into blastocysts.

Number of embryos at each stage per total two pronuclear (2PN) eggs (%), at Day 1-4 after IVF, produced using spermatozoa from males at 2 (n = 9) and ≥ 24 (n = 12) months of age, shown in Fig. 7c. Boxes, 1 cell (white), 2 cell (light gray), 4-8 cell (dark gray), morula (diagonal strokes), blastocyst (dots), and degenerated or fragmented embryos (black).

Supplementary Fig. 8

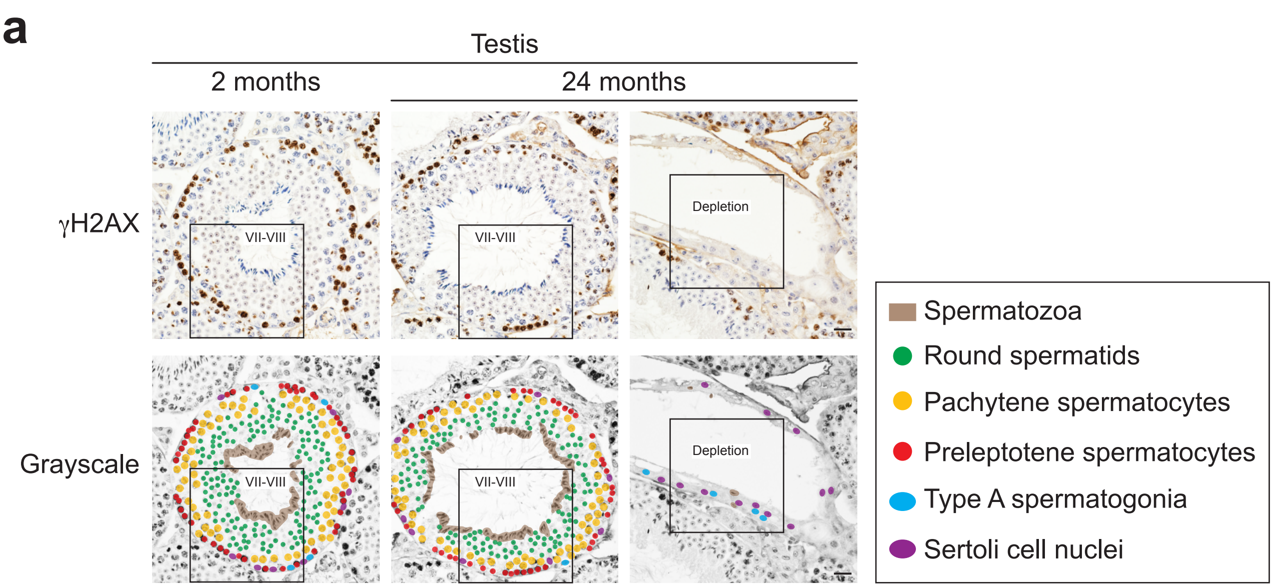

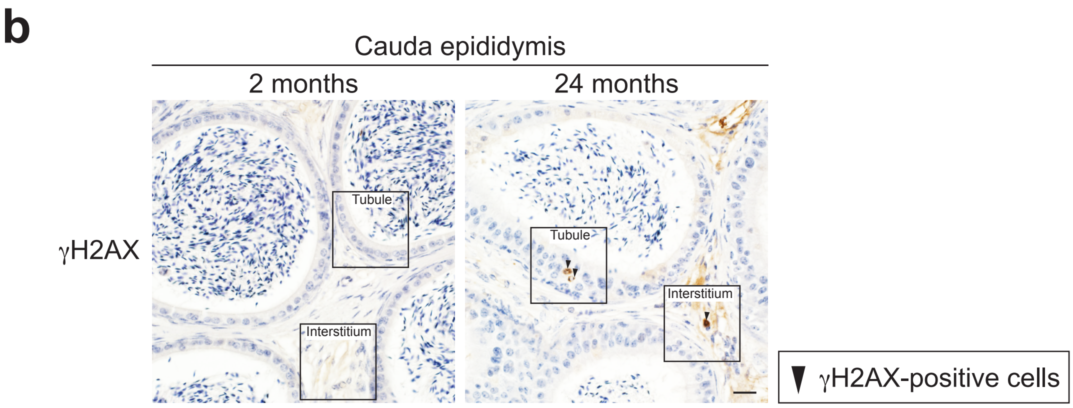

Supplementary Fig. 8 Somatic cells in aged epididymides, but not somatic or germ cells in aged testes, show detectable DNA damage signals.

(**a**) Immunostaining for γH2AX, with hematoxylin counterstain, on testis cross-sections of males at 2 (*Left*, a stage VII-VIII tubule) or 24 (*Middle*, a tubule in stages VII-VIII; *Right*, a tubule showing germ cell depletion) months of age. *Lower* panels are the grayscale versions of *Upper* panels. Dots, Sertoli cell nuclei (purple), type A spermatogonia (blue), preleptotene spermatocytes (red), pachytene spermatocytes (yellow), and round spermatids (green). Brown areas, spermatozoa. Black boxed regions are enlarged in Fig. 8a. Scale bars, 20 μm.

(**b**) Immunostaining for γH2AX, with hematoxylin counterstain, on cauda epididymis tubule longitudinal-sections of males at 2 (*Left*) or 24 (*Right*) months of age. Arrowheads, γH2AX-positive cells. Black boxed regions are enlarged in Fig. 8b. Scale bar, 20 μm.
