# Supplementary material for: Multiple ageing effects on testicular/epididymal germ cells lead to decreased male fertility in mice
Source: Commun Biol. 2024 Jan 4;7:16. doi: 10.1038/s42003-023-05685-2 (PMC10766604; doi:10.1038/s42003-023-05685-2)
Supplement: Supplementary file 3 — Description of Supplementary Materials [file 42003_2023_5685_MOESM3_ESM.docx]

**Description of Additional Supplementary Files**

**File name:** Supplementary Data 1

**Description:** Source data for fig 5a.

**File name:** Supplementary Data 2

**Description:** Source data for Fig. 5b and supplementary fig. S5

**File name:** Supplementary Data 3

**Description:** Source data for Fig. 5c_testis

**File name:** Supplementary Data 4

**Description:** The source data behind the graphs in the paper
