# Supplementary material for: Multiple ageing effects on testicular/epididymal germ cells lead to decreased male fertility in mice
Source: Commun Biol. 2024 Jan 4;7:16. doi: 10.1038/s42003-023-05685-2 (PMC10766604; doi:10.1038/s42003-023-05685-2)
Supplement: Supplementary file 8 — Reporting Summary [file 42003_2023_5685_MOESM8_ESM.pdf]

nature portfolio

Corresponding author(s): Tsutomu Endo and Masahito Ikawa

Last updated by author(s): Dec 6, 2023

Reporting Summary

Nature Portfolio wishes to improve the reproducibility of the work that we publish. This form provides structure for consistency and transparency in reporting. For further information on Nature Portfolio policies, see our Editorial Policies and the Editorial Policy Checklist.

Statistics

For all statistical analyses, confirm that the following items are present in the figure legend, table legend, main text, or Methods section.

n/a	Confirmed	
☐	☒	The exact sample size (n) for each experimental group/condition, given as a discrete number and unit of measurement
☐	☒	A statement on whether measurements were taken from distinct samples or whether the same sample was measured repeatedly
☐	☒	The statistical test(s) used AND whether they are one- or two-sided<br>*Only common tests should be described solely by name; describe more complex techniques in the Methods section.*
☒	☐	A description of all covariates tested
☐	☒	A description of any assumptions or corrections, such as tests of normality and adjustment for multiple comparisons
☐	☒	A full description of the statistical parameters including central tendency (e.g. means) or other basic estimates (e.g. regression coefficient) AND variation (e.g. standard deviation) or associated estimates of uncertainty (e.g. confidence intervals)
☐	☒	For null hypothesis testing, the test statistic (e.g. F, t, r) with confidence intervals, effect sizes, degrees of freedom and P value noted<br>*Give P values as exact values whenever suitable.*
☒	☐	For Bayesian analysis, information on the choice of priors and Markov chain Monte Carlo settings
☒	☐	For hierarchical and complex designs, identification of the appropriate level for tests and full reporting of outcomes
☒	☐	Estimates of effect sizes (e.g. Cohen's d, Pearson's r), indicating how they were calculated

Our web collection on statistics for biologists contains articles on many of the points above.

Software and code

Policy information about availability of computer code

Data collection	Sperm motility analysis: CEROS II sperm analysis system (software version 1.5.2; Hamilton Thorne Biosciences) and sperm motion analyzing software (BohBohsoft, Tokyo, Japan).
Data analysis	RNA-Seq analysis: TopHat version 2.0.13 (in combination with Bowtie2 version 2.2.0 and SAMtools version 0.1.18), Cuffnorm or Cuffdiff version 2.2.1, and Enrichr.<br><br>Statistical analysis: GraphPad Prism 9

For manuscripts utilizing custom algorithms or software that are central to the research but not yet described in published literature, software must be made available to editors and reviewers. We strongly encourage code deposition in a community repository (e.g. GitHub). See the Nature Portfolio guidelines for submitting code & software for further information.

Data

Policy information about availability of data

All manuscripts must include a data availability statement. This statement should provide the following information, where applicable:

- Accession codes, unique identifiers, or web links for publicly available datasets
- A description of any restrictions on data availability
- For clinical datasets or third party data, please ensure that the statement adheres to our policy

All data supporting the findings of this study are available from the corresponding authors on reasonable request.

Human research participants

Policy information about studies involving human research participants and Sex and Gender in Research.

Reporting on sex and gender	NO human research were involved in this study.
Population characteristics	See above.
Recruitment	See above.
Ethics oversight	See above.

Note that full information on the approval of the study protocol must also be provided in the manuscript.

Field-specific reporting

Please select the one below that is the best fit for your research. If you are not sure, read the appropriate sections before making your selection.

☒ Life sciences ☐ Behavioural & social sciences ☐ Ecological, evolutionary & environmental sciences

For a reference copy of the document with all sections, see nature.com/documents/nr-reporting-summary-flat.pdf

Life sciences study design

All studies must disclose on these points even when the disclosure is negative.

Sample size	Sample sizes were determined based on published studies in the field or our previous experiences. No statistics was used to predetermine the sample size.
Data exclusions	N/A
Replication	Technical or biological replicates are indicated in figure legends and methods. All replicates successfully reproduced the presented findings unless particularly stated. Otherwise, the number of replicates showing the presented findings are indicated.
Randomization	Animals were distributed at random over experimental groups.
Blinding	Investigators were not blinded to allocation during experiments.

Reporting for specific materials, systems and methods

We require information from authors about some types of materials, experimental systems and methods used in many studies. Here, indicate whether each material, system or method listed is relevant to your study. If you are not sure if a list item applies to your research, read the appropriate section before selecting a response.

Materials & experimental systems

n/a	Involved in the study
☐	☒ Antibodies
☒	☐ Eukaryotic cell lines
☒	☐ Palaeontology and archaeology
☐	☒ Animals and other organisms
☒	☐ Clinical data
☒	☐ Dual use research of concern

Methods

n/a	Involved in the study
☒	☐ ChIP-seq
☒	☐ Flow cytometry
☒	☐ MRI-based neuroimaging

Antibodies

Antibodies used

Anti-STRA8 (rabbit polyclonal, ab49405, Abcam)
Anti-BrdU (rat monoclonal; ab6326, Abcam)
Anti-SOX9 (rabbit polyclonal; AB5535, Millipore)
Anti-gH2AX (rabbit polyclonal; ab11174, Abcam)
Anti-OCT4 (rabbit polyclonal; PM048, MBL Life Science)
Anti-CDX2 (mouse monoclonal; B-MU392AUC, BioGenex)
Anti-rabbit IgG (horse polyclonal; ImmPRESS-HRP detection kit, MP7401, Vector Laboratories)
Anti-rat IgG (goat polyclonal; ImmPRESS-HRP detection kit, SK4105, Vector Laboratories)
Anti-rabbit IgG (goat polyclonal; DyLight 549, Thermo Fisher Scientific)
Anti-mouse IgG (goat polyclonal; DyLight 448,Thermo Fisher Scientific)

Validation

All antibodies were validated by previous papers and manufacturers.

Animals and other research organisms

Policy information about studies involving animals; ARRIVE guidelines recommended for reporting animal research, and Sex and Gender in Research

Laboratory animals

C57BL/6J mice were purchased from Charles River Laboratories Japan, Inc. and CLEA Japan, Inc.. Experiments were performed on male mice at 2, 6, 12, 18, 24, and ≥ 24 (from 24 to 26) months of age, and female mice at 2 months of age.

Wild animals

This study did not involve wild animals.

Reporting on sex

Sexes of animals are indicated in figure legends and methods.

Field-collected samples

This study did not involve field-collected samples.

Ethics oversight

All animal experiments were approved by the Animal Care and Use Committee of the Research Institute for Microbial Diseases, Osaka University, Japan.

Note that full information on the approval of the study protocol must also be provided in the manuscript.
